# Effects of Lipid Metabolites and Cross Organ Lipid Metabolism Regulation on Bone Homeostasis

**DOI:** 10.14336/AD.2025.0575

**Published:** 2026-06-08

**Authors:** Mengyuan Li, Jiaheng Zhang, Jiaqian Tang, Chang Zhou, Guomin Zhang, Huiping Liu, Xiaoming Lei

**Affiliations:** College of Integrated Traditional Chinese and Western Medicine, Hunan University of Chinese Medicine, Changsha 410208, Hunan, China

**Keywords:** osteoporosis, lipid metabolism, osteoblasts, osteoclasts, cross organ regulation

## Abstract

Osteoporosis is a metabolic bone disease characterized by the loss and destruction of bone microstructure. Its incidence rate increases with age and is an important public health problem. Recently, the relationship between lipid metabolism and osteoporosis has received widespread attention. Lipid metabolites, such as fatty acids, cholesterol, and adipose tissue-derived hormones, participate in maintaining bone metabolism homeostasis through key molecular networks that regulate bone cell function. In addition, the interaction between lipid metabolism and bone tissue involves cross-organ regulatory mechanisms, including the bone-adipose, bone-pancreas, and bone-gut axes. This study considers the types of lipid metabolites and their effects on bone cell function as a starting point for exploring the interaction between lipid metabolism and bone tissue. Furthermore, this study elucidated the specific mechanism of action of lipid metabolites on osteocytes, analyzed the relationship between lipid metabolism abnormalities and osteoporosis, and provided ideas for further clinical research on lipid metabolism-related osteoporosis.

## INTRODUCTION

Osteoporosis (OP) is a metabolic bone disease characterized by a reduction in bone mass and the destruction of the bone microstructure, which significantly increases the risk of fractures because of increased bone fragility. Owing to the aging of the global population, the incidence rate of OP is increasing annually and has become an important public health problem in the elderly. OP affects approximately 200 million people worldwide, and the number of female patients with OP is 4 times higher than that of male patients [1]. In China, the incidence of OP among people aged 65 years and older is as high as 32%, with females accounting for 51.6% of those affected [1]. OP not only seriously affects the quality of life of patients but also places a heavy economic burden on families and society.

Osteoporosis is characterized by an imbalance in calcium homeostasis, i.e., the dynamic balance between bone formation and resorption. This process is coordinated between bone formation, which is mediated by osteoblasts, and bone resorption, which is mediated by osteoclasts, to ensure the stability and functional integrity of the bone structure. Calcium homeostasis is crucial for bone development, repair, and maintenance of mechanical strength [2]. Studies have shown that lipid metabolites play an important role in the regulation of bone homeostasis. On one hand, lipid metabolites can directly act on bone cell signaling pathways, such as promoting osteoblast proliferation and differentiation by activating the Wnt/β-catenin signaling pathway [3]. On the other hand, lipid metabolism disorders may disrupt bone metabolism balance through multiple mechanisms, such as hypercholesterolemia, which can inhibit osteoblast activity and promote osteoclast differentiation, thus leading to reduced bone mass [4]. In addition, the abnormal accumulation of metabolites, such as cholesterol and triglycerides, in bone tissue can affect the differentiation direction of bone marrow mesenchymal stem cells (BMSCs) and alter the process of bone remodeling [5]. Research related to lipid metabolism has revealed an important cross-organ communication network between the gut, liver, adipose tissue, and bones. Strategies that target this inter-organ communication network (such as promoting bone formation by using gut microbiota metabolites or regulating adipokines to inhibit bone resorption) may restore the dynamic balance of bone homeostasis and synergistically enhance bone strength by coordinating metabolic, immune, and endocrine signals, thus providing innovative pathways for the systematic treatment of metabolic bone diseases.

This review delineates the lipid metabolite modulation of bone homeostasis, establishes its etiological role in OP, and reveals actionable therapeutic targets. Furthermore, a detailed description of the effect of cross-organ lipid metabolism regulation on bone homeostasis that highlights the influence of lipid metabolites on OP will not only help reveal the pathogenesis of OP but may also provide new strategies for clinical treatment, which have important scientific and clinical value.

### Direct Effects of Lipid Metabolites on Osteocyte Function

Lipid metabolites affect osteocyte function by regulating cell membrane characteristics, energy metabolism apthways including adenosine monophosphate-activated protein kinase (AMPK) and β-oxidation, and bone remodeling signaling axes: receptor activator of nuclear factor kappa-Β ligand (RANKL)/osteoprotegerin (OPG) and Wnt. Their effects are specific to metabolites and cell types and are intertwined with inter-organ hormone signaling, such as adiponectin and osteocalcin (OC).

#### Direct Regulation of Osteocytes by Fatty Acids

As primary lipid metabolites, fatty acids directly modulate osteocyte function through membrane incorporation and energy provision. Xiao et al. [6] studied the lipid metabolism of human adipose tissue stem cells and the number and activity of osteoblasts in CD36 knockout mice through clinical observations. Osteocyte membrane dynamics are driven by fatty acid signatures that directly determine cellular responses [7]. Different types of fatty acids have different effects on osteocytes. For example, polyunsaturated fatty acids (PUFAs) may promote the differentiation of mesenchymal stem cells into osteoblasts, whereas saturated fatty acids, such as palmitic acid, may have an inhibitory effect [8]. Fatty acids are an important energy source for osteocytes, which produce adenosine triphosphate (ATP) through β-﻿oxidation. This energy supply and the energy-sensing pathways it triggers, such as AMPK, are crucial for regulating osteocyte gene expression and function [8].

##### Direct Regulation of Osteocytes by Cholesterol

Cholesterol is an important product of lipid metabolism and plays multiple roles in regulating osteocyte function, particularly cell membrane stability and energy metabolism. Cholesterol is a crucial component of osteocyte membranes, and its abnormal metabolism can impair membrane function, thus subsequently affecting osteoblast activity and promoting osteoclast activation [9].

Cholesterol and its metabolites play important roles in energy metabolism in osteocytes. Mitochondria are the primary sites of cellular energy metabolism. Recent studies have found that a disruption in cholesterol metabolism may lead to mitochondrial dysfunction, which will affect the energy metabolism of osteocytes and ultimately result in impaired bone cell function [11]. This change in energy metabolism not only affects the normal physiological function of osteocytes but may also further disrupt the balance of bone metabolism by affecting osteoblast activity and osteoclast differentiation. For example, a high-cholesterol diet can increase the number and activity of osteoclasts, thus leading to a decrease in bone density [12]. Epidemiological studies have shown a positive correlation between hypercholesterolemia and the risk of OP [13]. Hypercholesterolemia is closely associated with the risk of osteoporotic fractures. According to data from the National Health and Nutrition Survey, approximately two-thirds (63.0%) of patients with OP suffer from hyperlipidemia, and the incidence of hyperlipidemia in elderly patients with OP is significantly higher than that in the general population [14]. The main mechanisms by which hypercholesterolemia disrupts bone homeostasis include the disruption of the balance of key signaling axes: RANKL/receptor activator of nuclear factor kappa-Β (RANK)/OPG that regulate bone resorption and the overactivation of osteoclasts. Inducing oxidative stress not only directly damages osteocytes but also inhibits osteogenesis, promotes osteoclast formation, and drives bone marrow stem cells to differentiate into adipocytes [14]. In high-cholesterol mouse models, experiments have shown that bone density decreases by 12.7%, bone trabeculae decrease by 38%, and the serum bone resorption marker TRAP5b increases by 2.3 times, thus confirming that oxidative stress is the core pathological mediator [16]. These pathways can be regarded as the key downstream executors of the negative effects of cholesterol. It is worth noting that cholesterol-induced inflammatory factors such as interleukin 6 (IL-6) and tumor necrosis factor alpha (TNF-α) not only directly act on osteocytes but also serve as important bridges connecting lipid metabolism abnormalities and bone-adipose axis dysregulation [17].

### Direct Regulation of Osteocytes by Adipocyte-Derived Hormones

#### Direct Effects of Adiponectin on Osteocytes

Adiponectin is a hormone secreted by adipose tissue and is widely involved in regulating energy metabolism, inflammatory responses, and tissue homeostasis. Adiponectin activates signaling pathways, such as AMPK, through its receptors, potentially promoting osteoblast differentiation and function and helping to maintain the balance between bone formation and resorption [19].

Under conditions such as obesity and aging, the body’s responsiveness to adiponectin decreases (adiponectin resistance), thus weakening the protective effects of adiponectin on bones[21]. Clinical observations have found a bidirectional relationship between the obesity phenotype and bone density; although increased mechanical load may enhance bone density (+8.3%, p < 0.05), adiponectin resistance caused by lipid metabolism disorders can counteract the protective effects of adiponectin on bone (odds ratio [OR] = 1.72, 95% confidence interval [CI] = 1.15-2.58) [22]. Adiponectin levels may also change during aging. In a mouse experiment, single-cell RNA sequencing showed that AdipoR1 expression in the bone marrow stromal cells of 16-month-old mice was reduced compared with that in 3-month-old mice, whereas AdipoR2 expression showed an upward trend during aging [23]. As age increases, the expression and function of adiponectin receptors (AdipoR1 and AdipoR2) may be inhibited, thereby altering their regulatory effects on osteocytes. However, the effects of adiponectin on bone cell function have been inconsistent. The role of adiponectin varies among different experimental models and clinical studies. The “bidirectional” effect of adiponectin on bone metabolism suggests that its effects are significantly context dependent (e.g., resistance in obese/aging states) and tissue specific. The activation of the AMPK pathway through receptors appears to be a core node in regulating energy metabolism and influencing the direction of BMSC differentiation (osteogenic vs. adipogenic). However, its net effect on bone resorption and formation may depend on its relative regulatory strength on multiple downstream pathways such as the OPG/RANKL axis, sphingosine-1-phosphate/C-X-C chemokine receptor type 4 axis, and peroxisome proliferator-activated receptor gamma (PPARγ). However, the hierarchy and interactions among these pathways require further clarification. Therefore, an in-depth study on the mechanism of action of adiponectin in osteocytes is important to understand its role in bone metabolism.

#### Direct Effects of Leptin on Osteocytes

Leptin, which is a pleiotropic adipokine, was originally characterized as an appetite regulator and was subsequently found to regulate bone metabolism. However, multiple studies have shown that leptin also plays an important role in bone metabolism and bone cell function, particularly in cell membrane function and energy metabolism. The cell membranes of osteocytes are key structures for material exchange and signal transduction in the external environment. Leptin binds to the corresponding sites on the bone cell membrane through its receptor, thus affecting ion channels and receptor activity on the bone cell membrane and regulating the intracellular calcium ion concentration[24]. This regulatory effect is crucial for maintaining the normal physiological function of osteocytes; for example, in osteoblasts, changes in calcium ion concentration can promote cell proliferation and differentiation. Research has found that a concentration of 2-10 mmol/L calcium ions can promote proliferation, osteogenic differentiation, and mineralization of human osteoblasts. Among them, 2, 4, and 6 mmol/L calcium ions can significantly promote the migration of human osteoblasts at 8, 16, and 24 hours, proliferation calcium ions significantly inhibit migration at 8 hours, but the mineralization induced by 8 and 10 mmol/L calcium ions is more pronounced. Additionally, high concentrations of leptin can alter the lipid composition of the cell membrane, thereby affecting the permeability and signal transduction efficiency. In chondrocytes, leptin-induced changes in the cell membrane may lead to the breakdown of the extracellular matrix, which in turn affects the phenotype of chondrocytes. In animal experiments, chondrocytes isolated and cultured in vitro were divided into a blank control group, a low concentration group of 10 ng/mL leptin, and a high concentration group of 100 ng/mL leptin. It was found that the expression level of matrix metalloproteinase-13 in the high concentration group was significantly higher than that in the blank control group, indicating that high concentration leptin can promote the breakdown metabolism of chondrocytes, induce mitochondrial autophagy damage, and promote the progression of osteoarthritis [25].

Energy metabolism is the foundation for osteocytes to maintain normal physiological functions, and leptin plays an important role in this process. Leptin activates the AMPK signaling pathway in osteoblasts, promotes energy metabolism, and supports the proliferation and differentiation of osteoblasts, thereby promoting bone formation. This mechanism of action indicates that leptin directly promotes the regulation of bone cell energy metabolism, which helps maintain the normal physiological functions of osteocytes [26]. Leptin has potential pro-osteogenic and anti-osteoclastic activities. This dual mechanism of action enables leptin to play a crucial role in maintaining the balance of bone metabolism, thus reflecting its complexity in regulating bone cell function [26].

Research has shown that leptin also plays a complex dual regulatory role in bone metabolism [27]. This dual regulatory mechanism is mainly achieved through the synergistic effects of the central nervous system (such as the hypothalamus) and peripheral tissues (such as osteocytes). On the one hand, leptin may inhibit bone formation through the hypothalamus, and the specific mechanism includes the activation of sympathetic nervous signals in the hypothalamus, thereby regulating the secretion of bone metabolism-related hormones and ultimately leading to a decrease in bone formation [28]. This central regulatory effect is important in maintaining energy balance throughout the body but may also have a negative effect on bone density [29]. Conversely, it directly targets BMSCs, stimulates osteoblastogenesis while suppressing adipogenic commitment, and could inhibit the differentiation of BMSCs into adipocytes. However, the net effect of leptin on human bone mass remains unclear. The relative strengths of its central inhibitory and peripheral stimulatory effects, as well as the effect of leptin resistance states, complicate the inference of precise causality from related research. Animal model studies have clearly demonstrated this bidirectional effect [30]. However, the results of human studies are not entirely consistent. Individuals who are obese often have hyperleptinemia and leptin resistance, which theoretically favor the central inhibitory pathway. However, epidemiological observations show that obesity is often associated with higher bone density (although fracture risk may increase because of an increased risk of falls), and this phenomenon is known as the “obesity paradox” in the field of osteology [31]. A simple causal relationship between leptin levels and bone mass is difficult to establish, and the net effect and clinical significance of leptin as a regulator of bone metabolism requires further in-depth research.

#### Direct Regulation of OC on Osteocytes

In recent years, significant progress has been made in the study of the endocrine functions of bone-derived factors. Xiaochun et al. identified 375 bone-derived factors with potential functions and established a dynamic network of bone-derived factors affecting bone remodeling and global homeostasis [32]. This study revealed the key roles of bone-derived factors, such as OC, lipocalin 2, and somatostatin, in systemic metabolic regulation. They not only participate in local bone metabolism but also act on distant organs through blood circulation to regulate glucose and lipid metabolism and energy balance. This section focuses on the functional exploration of OC.

**Figure 1.**
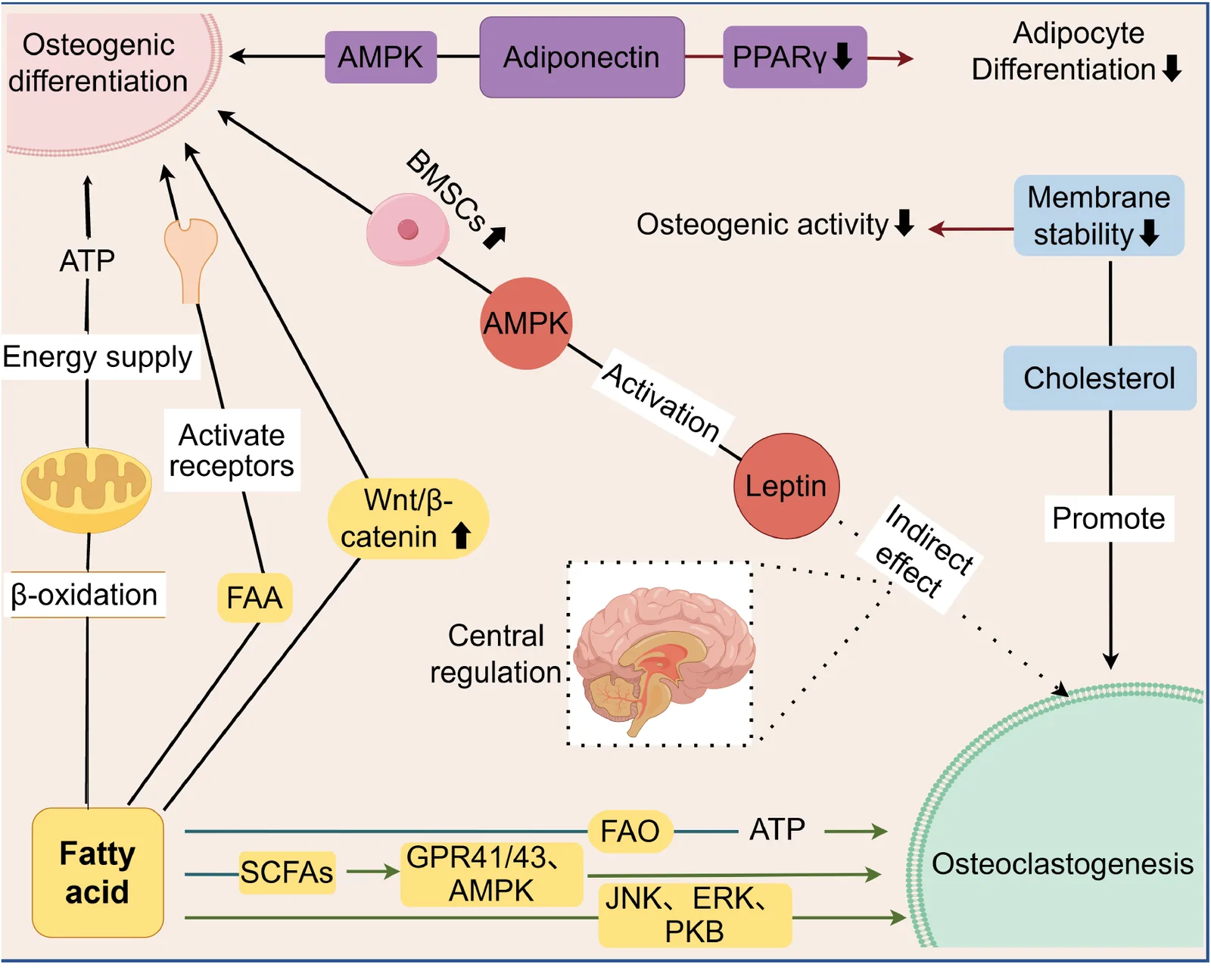
**The regulatory mechanism of lipid metabolites on bone cell function**. β-oxidation of fatty acids generates ATP for energy-intensive osteoblast differentiation via Wnt/β-catenin activation.; Its derivative FAA regulates osteoclast function by activating specific receptors. Cholesterol affects membrane stability and energy metabolism, inhibits osteoblast activity, and promotes osteoclastogenesis. Leptin activates AMPK signaling and regulates calcium ions, promoting bone cell proliferation and differentiation, and indirectly affecting osteoclast activity through the central nervous system. Adiponectin activates the AMPK pathway and regulates PPARγ activity, promoting osteogenic differentiation of BMSCs and inhibiting their adipogenic differentiation.

OC is a non-collagen protein secreted by osteoblasts and is mainly involved in bone mineralization. It is an important hormone that regulates bone metabolism, promotes bone formation, and inhibits bone resorption, thereby maintaining normal bone growth and development. OC exists in two forms: fully carboxylated OC and undercarboxylated OC (ucOC). OC, particularly ucOC, not only regulates bone mineralization but also serves as an endocrine factor that activates the adiponectin-PPARγ pathway through the GPCR6A receptor, thus promoting lipid oxidation in adipose tissue and insulin sensitivity [33]. Preclinical studies have shown that ucOC can improve atherosclerosis by inhibiting endothelial inflammation without vascular toxicity [34], thus making it a potential therapeutic molecule that targets both bone metabolism and cardiovascular disease. However, ucOC has low bioavailability in humans and is easily inactivated by carboxylation. Developing stable ucOC analogs or combining them with vitamin K antagonists (to inhibit carboxylation) may be the key to overcoming translational bottlenecks. It regulates insulin secretion and adiponectin expression through GPCR6A, establishing a direct evidence chain for the involvement of bones in the regulation of systemic glucose and lipid metabolism homeostasis and providing the strongest support for the concept of a “bone endocrine organ.” Its potential to improve atherosclerosis suggests that targeting OC signaling may simultaneously improve bone metabolism and cardiovascular metabolic risk, which has important translational medicinal significance. In summary, lipid metabolites profoundly affect osteocyte function by regulating cell membrane characteristics, core pathways of energy metabolism (AMPK and β-oxidation), and key bone remodeling signaling axes (RANKL/RANK/OPG and Wnt/β-catenin). Adipokines (adiponectin and leptin) and bone-derived hormones (OC) mediate the metabolic dialogue between organs, integrating bone homeostasis regulation into the systemic energy-lipid balance network. The AMPK pathway, which has the ability to integrate energy status with multiple lipid signals (fatty acid β-oxidation, adiponectin and leptin), and the RANKL/OPG axis, which plays a central role in directly determining the balance between bone resorption and formation, can be considered key hubs in this regulatory network. Understanding the hierarchy, such as AMPK as an upstream energy sensor that regulates downstream effector pathways, and interactions, such as cholesterol-induced reactive oxygen species inhibition of AMPK activity, between these mechanisms is crucial for identifying the core nodes of lipid metabolism disorders in OP. Targeting these hub pathways such as developing AMPK agonists and specifically regulating the RANKL/OPG ratio or key metabolites such as supplementing active ucOC, regulating specific fatty acid ratios, and interfering with cholesterol metabolism demonstrates potential therapeutic prospects but also faces translational challenges such as tissue specificity, signal complexity, and potential side effects (such as the metabolic effects of systemic AMPK activation) (Table 1 and Fig. 1).

### Effect of Lipid Metabolites on Osteocytes: Direct Actions and Bone Marrow Microenvironment

#### Direct Effects of Lipid Metabolites on Osteoblasts

Traditional research has focused on the utilization of glucose by osteoblasts; however, lipids and their derivatives are now considered important energy sources for osteoblasts. Fatty acids enter mitochondria through carnitine-mediated transport and undergo β-oxidation to generate ATP. Most studies have indicated that these fatty acids originate from stored triglycerides or adipose tissue and are released into the bloodstream through lipolysis. In addition, peroxisomes can also process some long-chain fatty acids, particularly during the differentiation stage of osteoblasts when their content increases, indicating the increased utilization of fatty acids as energy substrates [35]. There is a complex relationship between fat and bone. Although obesity is often associated with a low risk of fractures, recent research has revealed that certain factors released into the circulation from peripheral fat may negatively affect bone mass and increase the risk of fractures. Of particular importance is the local interaction between bone marrow fat and bone, which plays a crucial role in the pathogenesis of age-related bone loss and OP. This local interaction involves the autocrine and paracrine release of fatty acids and adipokines, which act on neighboring cells, including osteoblasts, thereby reducing their function and survival rates [36]. Osteoblasts express specialized receptors and enzymatic machinery for internalizing and catabolizing blood-borne lipids [37]. Pathological alterations in lipid homeostasis compromise bone-forming capacity, thus emphasizing the significance of this pathway. Kim et al. showed that these cells depend on external lipid sources to sequester lipids as inert energy reserves [38]. In terms of lipid uptake, osteoblasts express various lipid uptake receptors such as low-density lipoprotein receptor and CD36 and break down enzyme systems. CD36-ineffective mice have reduced bone mass because of impaired bone formation in osteoblasts[39]. This study further established bone tissue as a regulator of systemic lipid balance and showed that impaired lipid uptake by osteoblasts and osteocytes compromises metabolic homeostasis. For example, a deficiency in carnitine palmitoyl transferase 2 weakens the ability of osteoblasts to break down fatty acids, thereby affecting systemic lipid metabolism [39]. Fatty acid β-oxidation fuels osteogenesis, with anabolic signals necessitating enhanced mitochondrial oxidation. Calvarial osteoblast studies have confirmed that parathyroid hormone (PTH)/1,25-(OH)_2_D_3_ increases palmitate catabolism and augments mineralization [40]. Pathological states (aging, anorexia, and menopause) universally demonstrate inverse bones and adipose tissue dynamics in the marrow. The adipose cell-conditioned medium significantly inhibited osteoblast differentiation [41]. During osteoblast differentiation, a dynamic change occurs in lipid droplet accumulation, which provides fuel and supports the differentiation process [42]. However, the mechanisms underlying this decomposition require further investigation [43]. In addition, it is crucial to understand the different roles of lipids in the maturation stages of osteoblasts and the inhibitory mechanisms that reduce lipid droplet breakdown during maturation.

**Table 1:** Summary of Molecular Mechanisms of the Effects of Lipid Metabolites on Bone Cell Function.

Metabolites	Target cell type	Key signaling pathways	Functional effects	References
**Fatty acids such as DHA**	Osteoblasts	Wnt/β-catenin ↑	Promote osteogenic differentiation of MSCs↑	[8]
**cholesterol**	osteoclast	RANKL/OPG↑(imbalance)	Osteoclast differentiation↑/osteogenic activity↓	[14]
**Adiponectin**	BMSCs	AMPK/PPARγ ↑	Bidirectional bone formation ↓/bone resorption↓ (bidirectional regulation)	[19]
**Leptin**	Osteoblasts	AMPK pathway ↑	Osteogenesis↑/osteoclastogenesis↓	[27]
**Osteocalcin**	adipocyte	GPCR6A → Adiponectin ↑	lipid metabolism↑/bone strength↓	[31]

Various bioactive fatty acid derivatives, such as endocannabinoid-like compounds, exist within the bone tissue and may be locally synthesized and act on osteocytes [44].

Unsaturated fatty acids, particularly omega-3 PUFAs such as eicosapentaenoic acid (EPA) and docosa-hexaenoic acid (DHA), have beneficial effects on bone health [47]. They can be taken up by osteoblasts through specific receptors (such as free fatty acid receptor 4) and activate key signaling pathways (such as Wnt), thus promoting osteoblast differentiation and activity[48]. The mechanisms involve regulating the balance of the RANKL/OPG axis (inhibiting osteoclasts), reducing PPARγ expression (alkaline phosphatase activity, which is a marker of osteogenic differentiation, increases by 1.5-2 times, and the mineralized area increases by 30%-50% in vitro), and producing lipid mediators with anti-inflammatory and resorption-promoting activities [49]. Human studies have shown that supplementation with n-3 long-chain PUFAs can increase bone formation, affect peak bone mass, and reduce bone loss (the decrease in C-terminal telopeptide [CTX] levels is -0.367 μg/L [95% CI = -0.726 to -0.007]) [50]. Furthermore, n-3 fatty acids have been shown to help preserve bone mass in elderly women at risk of OP and improve bone quality in specific disease models [50].

The complications of bone healing, such as delayed healing and non-healing, affect approximately 5%-10% of patients with long bone fractures, thus leading to a significant decrease in the quality of life and an increase in medical expenses. The gut microbiota and its metabolites, particularly short-chain fatty acids (SCFAs), have been shown to have profound effects on almost all organs of the body, including the skeletal system. SCFAs demonstrate broad potential for promoting bone healing by directly regulating the function of key cells involved in fracture healing (osteoblasts, osteoclasts, chondrocytes, and fibroblasts) or indirectly mediating anti-inflammatory and immune regulatory responses. Given the regulatory role of SCFAs in the differentiation of osteoblasts and osteoclasts, they may also be involved in the integration of orthopedic implants and bone tissue. In addition, SCFA derivatives have been used in various tissue engineering architectures to reduce inflammation and induce bone tissue regeneration [51]. SCFAs persist in the fatty acid cycle and can be found in bone marrow serum, although their concentration and saturation vary [53]. In vitro and animal studies consistently demonstrated that long-chain saturated fatty acids (such as palmitate) inhibit osteoblast differentiation, whereas monounsaturated fatty acids (oleate) and n-3 PUFAs can alleviate this inhibition or directly promote osteogenic activity [54].

Cholesterol modulates osteoclastogenesis and cell viability as a critical cytoplasmic constituent. Experimental evidence has confirmed that monocyte membrane sterol levels drive multinucleation through the promotion of fusion [55]. 3-Hydroxy-3-methylglutaryl coenzyme A reductase inhibitors (e.g., simvastatin) suppress osteoclast differentiation in dose- and time-responsive patterns [56]. Studies have shown that high-density lipoprotein (HDL) can inhibit osteoclast activity and induce apoptosis by promoting cholesterol efflux and other mechanisms (HDL treatment at a concentration of 600 ng/mL can reduce osteoclast fusion index) [57], although the HDL-mediated osteoclast inhibition mechanism requires further investigation. Other studies have shown that the intermediates of the cholesterol biosynthesis pathway are crucial for the development of mature osteoblasts from mesenchymal stem cell (MSCs) [58]. The cholesterol biosynthesis pathway inhibitor atorvastatin inhibits mineralization by suppressing *ALPL* gene activity but does not affect OC expression [59].

#### Direct Effects of Lipid Metabolites on Osteoclast

Fatty acids critically modulate skeletal metabolism, particularly through the phenotypic programming of osteoclasts (differentiation, apoptosis, proliferation, activation, and remodeling). As exogenous receptor ligands, they govern intracellular transduction cascades (e.g., c-Jun N-terminal kinase/extracellular signal-regulated kinase/protein kinase B axis), ultimately reprogramming cellular bioenergetics [59]. This article delineates the direct contribution of fatty acids to OP pathogenesis, with a specific focus on their modulation of energy metabolism (e.g., glycolysis and oxidative phosphorylation). Exogenous fatty acids derived from adipocyte lipolysis, or de novo lipogenesis passively diffuse into osteoclasts where they are catabolized via fatty acid oxidation (FAO) to sustain bioenergetic homeostasis. Dodds et al. demonstrated that osteoclast differentiation is correlated with FAO upregulation. In support of this, the metabolomic profiling of plasma from 34 women revealed specific associations between serum CTX (a bone resorption biomarker) and lipid metabolic pathways, with no significant link to carbohydrate metabolism. The elevated FAO capacity observed in active osteoclasts implied a predominant reliance on lipid metabolism to fuel energy-demanding resorptive functions [61]. SCFAs such as propionate salts (C3) and butyrate salts (C4) inhibit osteoclastogenesis both *in vitro* and *in vivo*. On the basis of metabolic reprogramming (oxidative phosphorylation to glycolysis) and the downregulation of key osteoclast transcription factors, C3 and C4 treatments effectively prevented bone loss induced by ovariectomy (maintaining the trabecular volume fraction at ~80% vs. ~50% in the ovariectomy control group), which may be related to oxidative stress and acid accumulation [62]. Notably, under low-serum conditions (1% fetal bovine serum), osteoclasts upregulate FAO-related proteins, thus demonstrating FAO-mediated ATP production during osteoclastogenesis and resorptive activity [62]. Under these conditions, the synthesis of mitochondrial fatty acids (mainly saturated fatty acids) in osteoclasts increases; however, the specific mechanism remains to be further explored [63]. Notably, osteoclasts attached to bone fragments exhibited high FAO levels, although a glycolytic enzyme deficiency was also observed [64].

In addition to aging, atherosclerosis and OP share the pathogenesis of bone and vascular mineralization. Although the specific role of abnormal blood lipids (specifically elevated total cholesterol and low-density lipoprotein cholesterol) in this interaction is not well documented, MSCs are associated with low bone mass and an increased risk of fractures possibly via the exacerbation of oxidative stress and systemic inflammation, directly promoting increased osteoclast activity and reducing bone formation [65].

#### Direct Effects of Lipid Metabolites on BMSCs

In 1987, Fleisch et al. demonstrated that fatty acids constituted the dominant energy source for bone cells, and subsequent investigations identified these metabolites as secondary regulators (after glucose) of osteoprogenitor cell function [65]. In vitro experiments have shown that long-chain saturated fatty acids (such as palmitate) and certain omega-6 fatty acids (such as arachidonic acid) inhibit the osteogenic differentiation of BMSCs and may promote their differentiation into adipocytes[65]. Conversely, promoting fatty acid β-oxidation is beneficial for the osteogenic differentiation of BMSCs. BMSCs express specific fatty acids (such as GPR120), and their expression gradually increases with osteogenic induction [66].

In addition, childhood obesity may have adverse effects on bone development, thus leading to increased bone fragility, which is supported by reports of an increased incidence of limb fractures in obese children[67]. However, the bone mineral content of obese children is sometimes higher than that of normal-weight children, creating a complex picture. In obesity, multipotent BMSCs are more likely to differentiate into adipocytes than to osteoblasts, thus possibly affecting bone formation. In addition, adipocytes in the bone marrow release various pro-inflammatory and immunomodulatory molecules that promote the formation and activation of osteoclasts, thereby exacerbating bone fragility. Nevertheless, mechanical loading associated with obesity exerts an anabolic effect on bone accrual; therefore, the final quality and structure of bones are the result of a balance between inflammation and mechanical stimulation[67]. This framework merits a rigorous assessment to characterize MSC lipid utilization in osseous health and metabolic osteopathies.

Osteoblasts, osteoclasts, and BMSCs constitute the cellular basis of the bone tissue response to lipid metabolism, and their functions are mutually constrained, such as osteoblast-osteoclast coupling and BMSC differentiation decisions. Their energy status, receptor signaling, and differentiation fate are profoundly shaped by the local and circulating lipid environments. ﻿

### Molecular Mechanisms of Lipid Metabolism and Cross-Organ Regulation of Bone Tissue

In recent years, with the rapid development of molecular and systems biology, an increasing amount of evidence has revealed the molecular dialogue mechanisms between adipose tissue, bone tissue, and other key organs such as the liver, pancreas, and intestine. The interaction between lipid metabolism and bone tissue is not limited to local cellular signaling but also integrates the systemic metabolic status through a complex cross-organ regulatory network, thus achieving the coordinated maintenance of energy balance and bone health. On the basis of endocrine, neural, and immune mechanisms, multiorgan crosstalk regulates local tissue physiology while exerting global control over metabolic networks.

#### Bone-Adipose Axis

The crosstalk between bone and adipose tissues critically regulates lipid metabolism and skeletal balance. Adipokines (e.g., leptin and adiponectin) modulate bone remodeling through distinct pathways. Leptin modulates resorption and formation via sympathetic nervous system activation, and adiponectin indirectly affects bone metabolism by increasing insulin sensitivity. Factors secreted by bone tissue, such as OC, can also affect the function of adipose tissue; OC can regulate the metabolic function of adipose tissue, promote fat breakdown, and reduce serum triglyceride levels [68]. In addition, bone-derived PTH-related protein can increase energy consumption by inducing the browning of white adipose tissue [69]. It is worth noting that obesity, as a typical manifestation of abnormal lipid metabolism, has a particularly complex relationship with bone metabolism disorders[70]. Obesity-induced chronic low-grade inflammation disrupts the balance of the bone-adipose axis [71]. Elevated inflammatory factors (such as TNF-α and IL-6) not only directly stimulate osteoclast formation and inhibit osteoblast function but also alter the differentiation direction of bone marrow stem cells (toward adipocytes) and exacerbate bone loss in conjunction with other metabolic disorders, such as insulin resistance[72].

#### Bone-Pancreas Axis

Jiacan et al. found that bones secrete factors, such as OC and fibroblast growth factor 23 (FGF23), to regulate the pancreas across organs, thus forming an insulin-OC feedback loop and maintaining energy metabolism homeostasis [73]. Similarly, Ren et al. revealed the crucial role of the SLIT2 protein secreted by bones in fat metabolism, thus further confirming the importance of bones as endocrine organs in systemic metabolism[74]. These studies provide an important theoretical basis for the development of novel treatment methods targeting the bone-pancreas axis. The role of the bone-pancreas axis in lipid metabolism and energy balance is gradually receiving attention. Bones regulate their function across organs by secreting various bone-derived factors (such as OC and FGF23) within the pancreas, whereas insulin secreted by the pancreas also acts on the bones, forming a complex bidirectional regulatory mechanism.

OC is an important bone-derived hormone secreted by osteoblasts. The uncarboxylated form regulates insulin secretion and sensitivity. ucOC improves insulin resistance by activating GPRC6A receptors on pancreatic beta cells and promoting insulin secretion. In addition, ucOC indirectly affects insulin sensitivity by regulating adiponectin expression in the adipose tissue. This mechanism has been validated in a high-fat diet-induced obese mouse model, and studies have shown that enhanced ucOC activity can significantly improve systemic glucose homeostasis [75].

FGF23 is another key hormone secreted by osteocytes that primarily regulates phosphorus metabolism. However, abnormal FGF23 levels are also associated with insulin metabolic disorders and adipose tissue dysfunction and indirectly affect bone metabolism [76].

Insulin and glucagon, which are secreted by the pancreas, not only regulate blood glucose metabolism but also act on bones through endocrine pathways. Insulin promotes osteoblast activity and promotes bone formation. However, in the context of insulin resistance, leptin levels increase, thus inhibiting bone formation and further exacerbating the risk of OP. In addition, patients with chronic pancreatitis often have decreased bone density and an increased risk of fractures, which may be related to pancreatic exocrine dysfunction and vitamin D and K deficiency [77].

The discovery of the bone-pancreas axis provides a new perspective for understanding the pathogenesis of metabolic diseases, such as type 2 diabetes and OP [78]. For example, the fracture risk of patients with diabetes is significantly increased, but the bone density test results are often not reduced; this phenomenon is known as the “sugar bone paradox.” Research shows that advanced glycation end products (AGEs) play an important role in diabetes osteopathy, and AGE inhibitors may be a potential strategy for treating diabetes osteopathy [79].

#### Bone-Gut Axis

Owing to an in-depth study of the relationship between the gut microbiota and systemic metabolism, the role of the bone-gut axis in lipid metabolism and bone homeostasis regulation has gradually attracted attention. The gut microbiota and their metabolites affect bone metabolism through various mechanisms, including endocrine, immune, and nutrient absorption. The gut microbiota has a significant effect on bone metabolism through its metabolites (such as SCFAs and bile acids) and immune regulatory mechanisms. Research has shown that the disruption of the gut microbiota may lead to pathological bone loss in hosts, and the metabolites produced regulate bone metabolism through the gut-bone axis [80].

SCFAs, including acetic acid, propionic acid, and butyric acid, are the main metabolites produced during the fermentation of dietary fiber. SCFAs regulate bone metabolism through multiple mechanisms: inhibition of histone deacetylases (HDACs) and activation of G protein-coupled receptors (GPCRs) to suppress osteoclast differentiation. By contrast, butyric acid, which is an HDAC inhibitor, can inhibit osteoclast differentiation in bone marrow cells by regulating gene expression. SCFAs bind to GPCRs such as GPR41 and GPR43, thus inhibiting the development of osteoclasts [80]. By regulating the OPG and Wnt signaling pathways and improving nutrient absorption to promote osteoblast differentiation, a high-fiber diet increases SCFA levels, thereby increasing the solubility and absorption rate of calcium in the intestine and providing necessary nutritional support for bone formation[81]. By regulating immune cell balance and inhibiting inflammatory signaling pathways to improve inflammatory bone loss, SCFAs can regulate the balance of T helper 17 (Th17)/regulatory T (Treg) cells, inhibit the accumulation of pro-inflammatory cytokines, and alleviate inflammatory responses. This mechanism exerts significant protective effects against postmenopausal OP and inflammatory bone loss [82].

The gut microbiota affect bone metabolism by regulating the immune system. Th17 cells and Tregs play an important role in bone metabolism. Th17 cells promote bone resorption, whereas Tregs inhibit bone resorption. The gut microbiota affects bone remodeling by regulating the balance between these two cell types. It also regulates bone metabolism via neuroendocrine pathways. For example, the serotonin produced by the gut microbiota affects bone remodeling by regulating neurotransmitter levels. In addition, the gut microbiota affects bone homeostasis by regulating the secretion and action of PTH [83].

**Figure 2.**
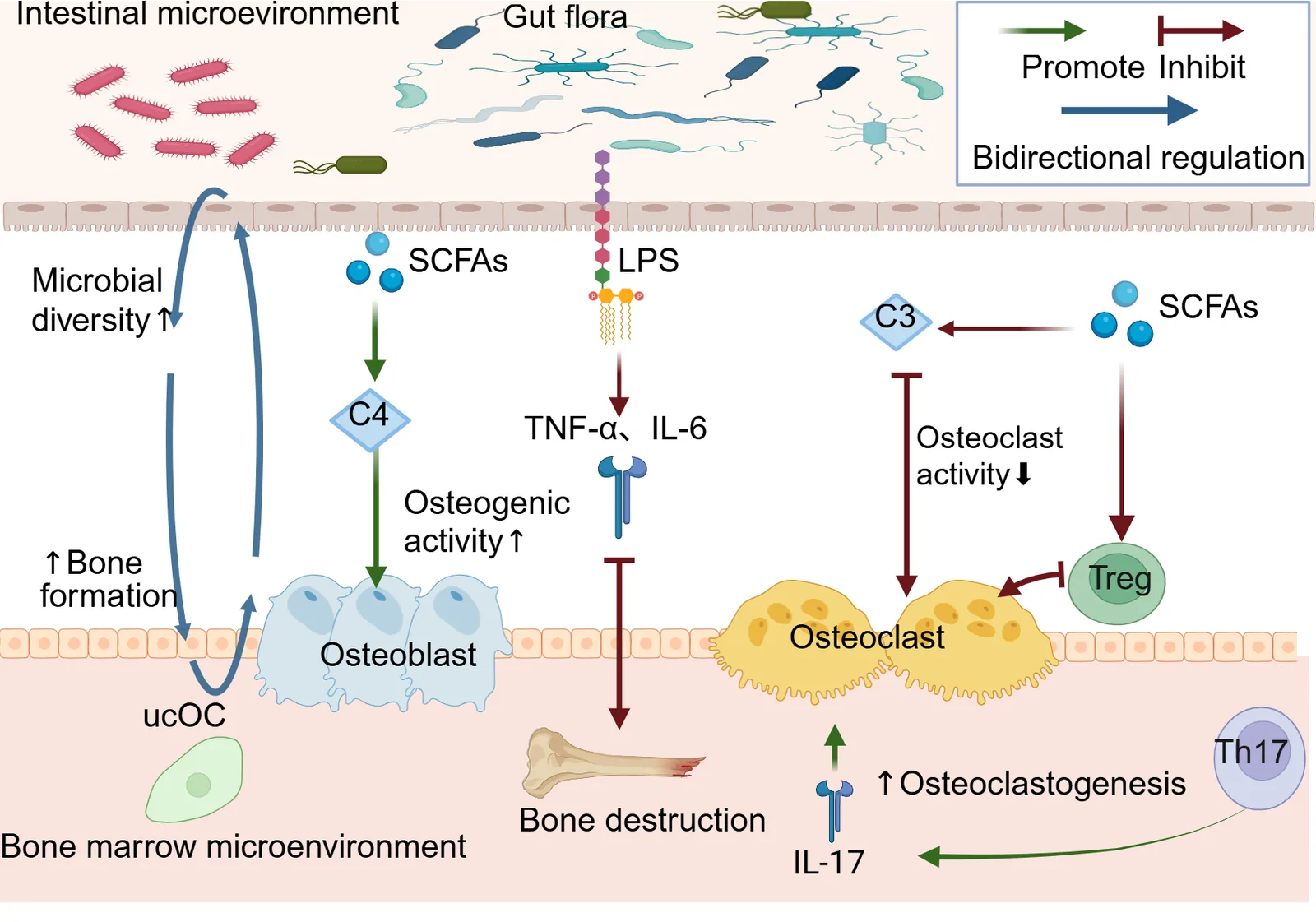
**The impact of cross-organ regulatory networks on bone homeostasis**. The propionic acid and butyric acid produced by the gut microbiota enter the circulatory system, with propionic acid inhibiting osteoclast activity and butyric acid promoting osteoblast activity. SCFAs induce Treg cell production and inhibit osteoclast activity. When the gut microbiota is imbalanced, LPS enters the bloodstream to activate TLR4, release inflammatory factors such as TNF-α and IL-6, disrupt bone homeostasis, and IL-17 secreted by Th17 cells promotes osteoclast differentiation. ucOC secreted by bones regulates the diversity of gut microbiota.

The bone-gut axis regulates lipid metabolism and bone homeostasis via various molecular mechanisms. The gut microbiota and its metabolites (such as SCFAs and bile acids) have a significant effect on bone metabolism through the endocrine, immune, and nutrient absorption pathways. This cross-organ regulatory mechanism not only provides a new perspective for understanding the pathogenesis of bone metabolic diseases but also offers potential targets for developing novel therapeutic strategies. Future research should further reveal the complex mechanisms of this axis and explore its potential clinical applications (Table 2 and Fig. 2).

The bone-adipose, bone-pancreas, and bone-gut axes collectively weave a systemic regulatory network for lipid metabolism and bone homeostasis. Within this network, the gut microbiota and its metabolites (such as SCFAs) are at the input end of the regulatory network. Multiple hormonal factors secreted by bones, adipose tissues, and the pancreas (such as OC, leptin, adiponectin, insulin, and FGF23) serve as core mediators of inter-organ communication. These signals ultimately converge within the bones and regulate key nodes, such as the core bone remodeling signaling pathway (e.g., RANKL/RANK pathway), cellular energy status sensing pathways (e.g., AMPK), and inflammatory responses, thus collectively determining the balance between bone formation and resorption. The current research challenge lies in dissecting the relative contributions and dynamic interactions of these axes under specific physiological and pathological conditions (such as aging, obesity, and menopause). Are there synergistic effects between the interventions across different axes? Translational applications must consider the targeting, individualization, long-term safety, and efficacy of the interventions.

### Therapeutic Strategies Targeting Lipid Metabolism for OP

Regarding pharmacological interventions, the potential effect of statins on OP requires comprehensive consideration. Although they exhibit bone-protective effects through cholesterol reduction and potential bone-forming actions in animal models, clinical evidence is highly contradictory. Observational studies suggest a reduced risk of fractures, whereas large randomized controlled trials (RCTs) have not consistently reported their effects. The core limitations are the insufficient exposure of bone tissue to conventional doses, confounding factors (such as health behavior bias), and risk of long-term myotoxicity. Therefore, they are not recommended as a first-line treatment for OP and require validation through dedicated RCTs [83]. AMPK agonists (such as metformin) have bone-protective effects in diabetic OP models; however, clinical research results are inconsistent (effective in small-scale RCTs but negative in large studies) [84]. Novel specific agonists (such as A-769662) require bone-targeted delivery systems because systemic activation interferes with glucose and lipid metabolism [85]. In terms of dietary intervention, although n-3 PUFAs (EPA/DHA) inhibit the RANKL/nuclear factor of activated T cells 1 pathway and reduce bone marrow fat deposition mechanistically, RCT evidence is highly inconsistent: supplementation with 1 g/day EPA + DHA in postmenopausal women can increase lumbar bone mineral density by 1.8% annually (p < 0.05). However, most RCTs have not shown significant improvement in bone turnover markers, and efficacy is influenced by baseline n-6/n-3 ratio and metabolic phenotype [86]; gut microbiota modulation, as an emerging intervention strategy, has the advantage of high safety and good compliance. Probiotics/prebiotics (such as lactobacillus and inulin) inhibit osteoclast activity by increasing SCFAs (35% reduction in tartrate-resistant acid phosphatase-positive cells), and small-scale RCTs show a slowdown in annual decline in hip bone mineral density by 0.7% (compared with 1.5% in the control group); however, the effect is limited by individual differences in microbiota, and ﻿SCFA bone marrow concentration is only one-tenth of that in the gut [87]. The treatment of OP should consider individual differences comprehensively and combine pharmacological, dietary, and lifestyle interventions to develop individualized treatment plans.

**Table 2:** Cross-organ axes regulating bone homeostasis through lipid metabolites.

Axis	Key factors	Regulatory mechanism	The impact on bone homeostasis	References
**Bone-adipose axis**	Leptin, adiponectin, osteocalcin	Sympathetic nervous system activity↑→Lipolysis↑	Leptin: bone formation↓, adiponectin:bone formation↑	[70]
**Bone-pancreas axis**	Osteocalcin (ucOC), FGF23	Insulin secretion↑/sensitivity↑	(Improve insulin resistance) bone formation↑	[76]
**Bone-gut axis**	SCFAs, bile acids	Treg cells ↑→ osteoclast activity ↓; ﻿calcium absorption ↑	Osteoclasts↑/osteoblasts↓	[79]

### Conclusion and Prospect

This article reviews lipid metabolite varieties and their influence on bone cells by focusing on the regulatory crosstalk at the lipid-bone interface. It specifically analyzes how these metabolites modulate osteoblasts, osteoclasts, and BMSCs while clarifying the systemic effects of inter-organ lipid regulation on skeletal homeostasis. Evidence indicates that lipid metabolites orchestrate bone cell activity via diverse molecular pathways, maintain metabolic equilibrium, and represent novel therapeutic avenues for OP.

Although there has been some progress in research on the relationship between lipid metabolism and OP, there are still some shortcomings and challenges. First, the specific mechanism of action of lipid metabolites on bone cell function is not fully understood, particularly the regulatory mechanisms under different pathological conditions, which require further research. Second, more clinical and experimental data are required to support the causal relationship between abnormal lipid metabolism and OP. In addition, the cross-organ regulatory network of lipid metabolism and bone tissue is complex and involves interactions between multiple organs and systems. Clarifying the net effects and mechanisms of key metabolic factors (such as leptin) on bone homeostasis in complex physiological environments (such as obesity and aging) is an important prerequisite for understanding lipid metabolism-bone axis regulation ﻿and developing effective intervention strategies. According to the mechanism network integrated in this study, prioritizing the targeting of key hub pathways (such as developing tissue-specific AMPK modulators and precise intervention of RANKL/OPG balance) or core organ axes (such as supplementation with ucOC, regulation of the bone-gut axis using SCFAs/probiotics, and utilization of the pleiotropic effects of statins) holds great promise. Combined intervention strategies (such as anti-inflammatory agents, bone formation promotion, and microbiota modulation) may yield synergistic effects. Rigorously designed clinical trials are urgently needed to evaluate the efficacy, safety, and individualized applicability of these targeted strategies for different types of OP (postmenopausal, senile, glucocorticoid-induced, and diabetes-related OP). The utilization of multi-omics technologies (metabolomics and microbiomics) to identify novel biomarkers reflecting the status of the lipid metabolism-bone axis will aid in the early identification of high-risk populations and guide precise treatment. As our understanding of the integrated regulatory network of lipid metabolism and bone homeostasis deepens, the modulation of lipid metabolism is expected to become an effective new strategy for preventing and treating OP, thereby improving patients’ quality of life.

## Data Availability

Data availability is not applicable to this article as no new data were created or analyzed in this study.
